# A postmenopausal woman with sciatica from broad ligament leiomyoma: a case report

**DOI:** 10.1186/s13256-016-1089-y

**Published:** 2016-10-31

**Authors:** Ya-Chu May Tsai

**Affiliations:** Department of Medicine, St Vincent’s Hospital Melbourne, 41 Victoria Parade Fitzroy, 3065 Melbourne, VIC Australia

**Keywords:** Broad ligament leiomyoma, Case report, Leiomyoma, Sciatica

## Abstract

**Background:**

Unilateral lower abdominal pain and/or sciatic nerve pain is a common presentation in the elderly population. The prevalence of broad ligament leiomyoma is <1 % with the prevalence declining after the menopause and it is rare for broad ligament leiomyomas to be clinically significant. Thus, we highlight a case of symptomatic broad ligament leiomyoma in a postmenopausal woman whose symptoms improved after definitive treatment.

**Case presentation:**

A 62-year-old postmenopausal Macedonian woman was referred to our gynecological department with unexplained pain in her left leg and left iliac fossa region on walking. There was minimal relief with increasing analgesia use prescribed by the family physician. Investigations revealed an ipsilateral adnexal mass and subsequent treatment with laparoscopic broad ligament myomectomy helped to alleviate her symptoms.

**Conclusions:**

Our case highlights the importance of staying mindful of alternate diagnoses when presented with a common presentation of iliac fossa pain and pain in the leg. Although broad ligament leiomyomas are benign tumors, the uncommon symptomatic presentation led us to report and focus some attention on this type of tumor.

## Background

Pathology of the broad ligament is rare and the prevalence of broad ligament leiomyoma is <1 % [1]. Leiomyomas (fibroids) are benign tumors of the uterus affecting 30 % of women of reproductive age and its prevalence declines after the menopause [2]. While leiomyomas are the most common female genital neoplasm, 50 % of women affected are asymptomatic and it is rare for broad ligament leiomyomas to be clinically significant [3].

## Case presentation

A 62-year-old Macedonian secundipara woman was referred to our gynecological department for 2-year history of unexplained left iliac fossa pain and pain in her left leg on walking. Our patient was 15 years postmenopausal with no history of hormonal replacement therapy use. There had been no symptoms referable to the bladder or bowels or abnormal per vaginam bleeding. Our patient’s family and surgical history was unremarkable. The family physician has attributed her pain to lumbar canal stenosis based on clinical examination and subsequently prescribed increasing doses of analgesia with minimal effect.

On physical examination, there was minimal abdominal tenderness. A speculum examination showed atrophy consistent with postmenopausal status. A bimanual pelvic examination revealed a non-tender, mobile mass in the left adnexal region with no other signs suggestive of malignancy.

### Investigations/treatment

Her serum level of cancer antigen (CA)-125 was 5 U/mL. A computed tomography (CT) scan of her abdomen and pelvis revealed a solid well-defined mass in the left adnexa. Her liver, pancreas, spleen, and adrenal glands were normal and no ascites or lymphadenopathy was noted. There was lumbar canal stenosis at L4 and L5 levels, more severe on the right than the left, though this did not seem to explain our patient’s symptoms. Following the CT scan findings, a pelvic ultrasound (US) was organized and showed a 9 × 6.3 × 4.9 cm solid mass in the left adnexal region.

Our patient underwent laparoscopic myomectomy; the mass originated from the left broad ligament of the uterus. Intraoperatively, there was no connection among the uterus, the left ovary, and the mass. On pathologic examination of the specimen, the tumor was diagnosed as a large 93 × 58 × 40 mm leiomyoma with no significant surrounding non-neoplastic tissue.

## Discussion

Our patient is of interest for a number of reasons. Her age of 62 years with postmenopausal status is unusually late for patients with broad ligament or other gynecologic leiomyomas. In a postmenopausal woman, the presence of an adnexal abnormality raises the possibility of malignancy such as leiomyosarcoma [3] or ovarian cancer [4]. A broad ligament leiomyoma is not usually in the differential diagnosis of an adnexal mass.

Further, symptoms of leiomyoma are typically those of abnormal uterine bleeding, menstrual disturbances, pelvic pain, and pressure symptoms such as bloating, urinary frequency or bowel disturbances [2]. Unilateral lower abdominal pain or sciatic nerve pain, such as in our patient is said to be extremely rare [5, 6]. While our patient did have a positive history of lumbar canal stenosis, she reported alleviation of her symptoms after surgery, suggesting the leiomyoma to be causing, if not at least contributing, to her sciatica.

Surgically, laparoscopic broad ligament myomectomy is difficult to perform for leiomyomas lying low on the sacrum as these may receive sacral blood supply. Although prospective randomized trials have shown that complication rates for laparoscopic myomectomy are comparable to abdominal myomectomy [7, 8], these trials were based on premenopausal women with uterine myomectomy. More research into the risks of broad ligament myomectomy, either laparascopic or abdominal, is needed to better inform patients about the potential surgical complications of such procedures. Our patient reported alleviation of symptoms upon postoperative review in clinic.

## Conclusions

Unilateral lower abdominal pain or sciatic nerve pain is a common presentation in the elderly population. Although broad ligament leiomyoma is a rare cause of such presentation, our case highlights the importance of staying mindful of alternate diagnoses.

### Patient’s perspective

Postoperative course was uneventful and at 6 months postoperative review, she reported alleviation of her symptoms after surgery. Our patient was grateful for the investigations and procedures performed. She felt she was listened to and was not just “another lady with chronic back pain”.
